# Functional Symptoms in Organic Nerve Injuries

**Published:** 1918-09

**Authors:** 


***So-Called Functional Symptoms in Organic Nerve Injuries.***
By John S. B. Stopford. Abst. of a Report to the Medical Research Committee. *The Lancet*, June, 8, 1918.

The author states that not uncommonly functional symptoms accompany undoubted organic injuries to the peripheral nerves. As Babinski suggests, this functional defectiveness may result from faulty operation and treatment, but the author believes that a large portion of such cases are due to a mixture of organic, psychic, and functional defects.

When the functional defect is the predominating symptom, there is danger that the weakness be attributed exclusively to hysteria when in reality an organic injury exists; hence the necessity to investigate the faradic response of all the affected muscles. Often there is a slight thermalgia in such instances.

When there are manifest organic symptoms the diagnosis is simpler. In one case a partially severed median nerve was found to be involved in the scar, causing neuralgic symptoms when there was a forcible extension of the forearm.

Pain is a frequent localizing factor, but it should be remembered that where there is an organic defect the pain itself may sometimes be psychic.

The author regards an irritative organic element to be an almost constant attendant in these cases. Failure to obtain good functional results is frequently due to the neglect of this defect. Cases which stubbornly fail to react to treatment or soon relapse are probably those in which organic disturbance persists.

Somatic trauma undoubtedly plays a larger part in such cases than is generally supposed. It is often the exciting cause. There can be little doubt, however, that the predisposing cause is of psychogenic origin. Even those with no previous neuropathic history may be overcome by the nerve strain of battle and so develop hysterical functional disturbances.

The first aim of any method of treatment should be to establish positively whether there is present any somatic trauma. When once this defect is remedied, psycho-therapic methods of treatment should have every chance of success. In proceeding to this branch of the treatment, it is well to take the patient completely into one's confidence and to explain how the limb has been " forgotten " and how " consciousness " of it may be regained. It is advantageous to deal with sensory loss first, and this can be done most effectively by means of a faradic current with a brush electrode. This is a helpful preliminary to functional recovery.

In regaining voluntary movement, the patient should first have his attention fixed upon a simple movement, as extension or flexion, on the unaffected side. While this simple movement is being passively performed on the affected side, his attention should be transferred; as progress is often slow, the patient should have all possible help and encouragement. It is well to give him some light form of employment which calls for the exercise of the function in process of restoration. In no case should the limb in the meantime be immobilized.

Environment and emulation are important aids not only in recovery but in prevention of relapse. As one of the most frequent causes of relapse is psychical exhaustion, every effort should be made to give employment that would not expose the patient to emotional disturbance.

